# Measures to Protect the Health and Safety of Massachusetts Employees Who Must Work at the Workplace During the SARS-CoV-2 Pandemic

**DOI:** 10.1177/1048291120960229

**Published:** 2020-09-22

**Authors:** Jodi Sugerman-Brozan, Health Technical Committee of the Massachusetts Coalition for Occupational Safety and Health

**Affiliations:** 1 Massachusetts Coalition for Occupational Safety and Health, Boston, MA, USA

**Keywords:** COVID-19, economic reopening, Massachusetts, worker health and safety

## Abstract

The Massachusetts Coalition for Occupational Safety and Health (MassCOSH) developed workplace health and safety recommendations for Phase 2 of the Massachusetts plans to reopen the economy as the spread of SARS-CoV-2 novel coronavirus was reduced in the state. The governor’s plan included minimal measures for workplace health and safety protections during this pandemic. The MassCOSH recommendations are presented in this document.

## Introduction

The Massachusetts Coalition for Occupational Safety and Health (MassCOSH) is a forty-four-year-old nonprofit organization dedicated to ensuring that all workers can go to work, earn a fair wage, be treated with respect and dignity, and return home to their families alive and well. MassCOSH is a member of the National Council for Occupational Safety and Health and the recommendations listed below were informed not only by the local experts who are members of its Health Technical Committee (certified industrial hygienists, occupational health physicians, academic researchers, attorneys, epidemiologists, and other occupational health specialists) but also by a task force of national workplace health and safety experts.

On 11 May 2020, MassCOSH presented Massachusetts Governor Charles Baker and his appointed Reopening Advisory Board with a set of recommendations for workplace health and safety measures required for the phased reopening of Massachusetts workplaces if and when the spread of the SARS-CoV-2 novel coronavirus was steadily contained within the state.

The following document was publicly issued by MassCOSH on 3 June 2020. The recommendations for protecting workers during the pandemic were supported by the Massachusetts AFL-CIO and adopted by two coalitions organizing for a healthy, safe, and just response and recovery from the pandemic: The Massachusetts COVID 19 Response Alliance and the Emergency Task Force on Coronavirus & Equity (convened by the Massachusetts Public Health Association). As of late August 2020, Massachusetts Governor Baker has made minimal progress toward implementing most of these recommendations, choosing instead to rely on under-resourced local health officials for enforcement, essentially deferring to the judgment of the state’s employers when it comes to workplace measures to protect workers during this pandemic.


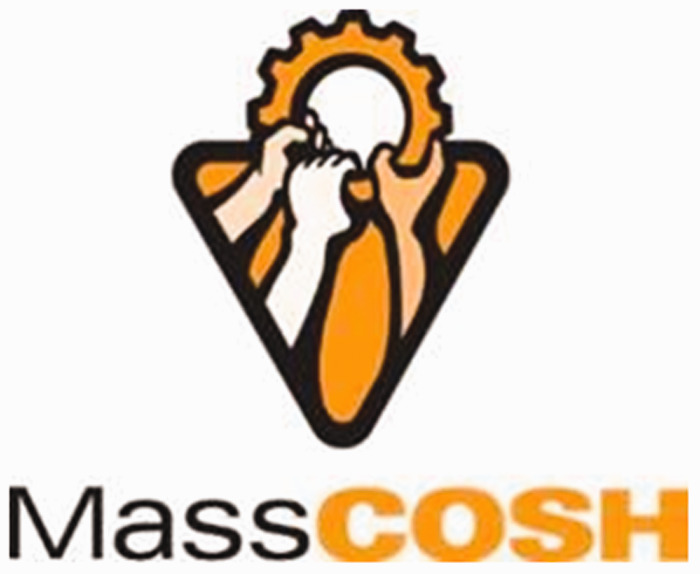


## Workers Are Not Expendable Commodities

### Reopening Massachusetts Phase 2 Demands

**3 June 2020**

On May 18, MassCOSH issued failing grades to Governor Baker’s Reopening Massachusetts plan because of its failure to adequately protect workers and the public. Now, as the state quickly moves toward Phase 2 in which restaurants, childcare facilities, retail stores, hotels, libraries, and some movie theaters will be opening, thousands of workers will be heading back to their jobs. Yet, essential and Phase 1 workers continue to contract COVID-19 every day. MassCOSH has updated its *COVID-19 TOOLKIT FOR WORKERS: Health and Safety Protections and How to Make Them Happen* to help support these workers to organize for the protections they deserve. It can be found here. *Workers are not expendable commodities. They must be protected from exposure to the virus. Their lives literally depend on it, and so do ours*.

On May 11, MassCOSH released a set of recommendations to the Reopening Advisory Board most of which went unheeded. Based on these recommendations, our demands before Phase 2 of the Reopening Massachusetts Plan are as follows:

- Stronger, enforceable health and safety protections for workers that include workplace-specific COVID-19 safety plans designed in collaboration with workers. In particular, protections to address aerosol transmission of micro-droplets of the virus are needed.
- Resources and technical support for the Local Boards of Health (LBOH) that have been given the job to enforce the state’s Mandatory Safety Standards for Workplaces. Furthermore, the state must revise any guidance that undermines LBOH’s authority to apply higher safety standards and/or close businesses that they believe are endangering workers and the public.
- Protection of workers’ voices and workers’ rights to information, to report and refuse dangerous work without retaliation, to job retention, and to pay or benefits if they are at high risk and cannot work. For workers who do become sick as a result of workplace exposure, Workers’ Compensation benefits should be mandated, using a conclusive presumption.
- Workplace exposure is a major way our residents are becoming ill and spreading the virus to their families and communities. Before we move to Phase 2, we must begin collecting and analyzing data on the occupation and industry of COVID-19 cases, and develop procedures to investigate workplace outbreaks and close businesses due to COVID-19 infections and outbreaks when needed.

More details on how to meet these demands are given below. As we refer to workers, we mean *all* workers. This means anyone who performs labor, including full-time and part-time, private, public, and nonprofit sectors, permanent and temporary, independent contractors (including workers often referred to as “gig” workers), and employees of subcontractors or staffing or temp agencies.

Stronger, enforceable health and safety protections for workers that include workplace-specific COVID-19 Safety plans designed in collaboration with workers.

The reopening plan’s Mandatory Safety Standards for Workplaces focus on recommendations about preventing the spread of the virus through social distancing, hygiene protocols, staffing and operations, and cleaning and disinfecting. While these measures are necessary, they are not sufficient. Employers provide protections that align with the National Institute of Occupational Safety and Health (NIOSH) “hierarchy of controls” that favors more protective elimination, substitution, and engineering controls over less protective administrative controls and personal protective equipment. Specifically, before Phase 2, standards should be added that require:

- Involvement of workers and unions in the process of creating workplace-specific COVID-19 Safety Plans, or health and safety procedures. The state abandoned essential workers for the first two months of this crisis, leaving them to fend for themselves with no protective standards in place. Then, it excluded them and their organizations from the Reopening Advisory Board. Now, their voices and the voices of all workers are missing from the plan, its industry-specific standards, and their implementation.
- **Scheduling and staffing** decisions and assignments designed to minimize exposure.
- **Personal Protective Equipment (PPE)**, assessments of the necessary respiratory protection, fit testing as needed, and sanitizing and storage of respirators. Employers must provide all appropriate PPE without requiring employees to improperly reuse equipment.
- **Protections to address aerosol transmission of micro-droplets** of the virus, which can travel distances of more than six feet, can linger in the air for significant periods of time, and can be inhaled by someone wearing a cloth face mask. These protections would include requirements for proper ventilation and adequate PPE.
- **Training** for all workers, in their preferred language, that includes not only social distancing and hygiene protocols but also basic rights on the job (including available benefits), proper donning and doffing for PPE, and general information on routes of exposure.
- The Safety Standards for Workplaces should require that all employers, not just some, designate a COVID-19 health and safety officer.
- **All businesses maintain a log of all employees, visitors, vendors, and contractors visiting the premises** every day; in order to support contact tracing efforts.
- **Mandatory compliance with the standards.** The standards are often only required “if feasible” or “when possible” giving employers plenty of opportunities to avoid compliance.

**Resources and technical support for the LBOH that have been given the job to enforce the state’s Mandatory Safety Standards for Workplaces. The state must revise any guidance that undermines LBOH’s authority to apply higher standards and/or close businesses that they believe are endangering workers and the public.**

- Enforcement of the existing workplace standards is given to LBOH and the Department of Labor Standards (DLS). Neither of them have the staff or resources to enforce these protections on the scale necessary. Funds must be allocated to provide **additional resources** to both DLS and LBOH in order to hire/train inspectors and conduct inspections, analyze data, and so on. The state Department of Public Health should provide training and technical assistance to LBOH.
- This includes no planned or targeted inspections of workplaces and leaves workers (and customers) to notify authorities if there are unsafe conditions. In addition to having employers “self-certify” that they are meeting workplace health and safety standards, LBOHs should have the option to **conduct inspections when they deem necessary.**
- All employers must post information with a multilingual hotline for workers to call and report unsafe conditions and seek support. Attorney General Maura Healey’s office has stepped up to provide this for workers. They take complaints and make referrals to the appropriate enforcing agency.
- LBOH and DLS must have **more flexibility in issuing fines and closing noncomplying businesses**. Guidance given to municipalities requires escalating enforcement. Penalties to employers who don’t follow standards are minimal and are only issued after verbal and written “redirection” is ignored twice. A cease and desist letter is only issued after the employer has been redirected up to five times. When workers and the public are in danger of exposure, LBOH and DLS must have the authority to move more quickly and decisively. Employers who fail to implement appropriate protective measures and expose workers to the risk of COVID-19 are assessed commensurate civil and criminal penalties for noncompliance. Furthermore, current guidance also requires that LBOHs or DLS must now first seek a court injunction whereas before they could issue a cease-and-desist order on their own authority. This court injunction requirement must be removed before Phase 2. The standard should ensure that LBOHs have the ability to raise the standards set by the state in their own cities and towns. The guidance states that cities and towns should not adopt stricter-than-the-Commonwealth-of-Massachusetts rules or ordinances that are intended to address the risks of COVID-19. *Cities and towns must have the ability to set their own standards that exceed the state standards*, especially if the state has not set adequate health and safety standards.

**Protection of workers’ voices and workers’ rights to information, to report and refuse dangerous work, to job retention without retaliation, and to pay or benefits if they are at high risk and cannot work. For workers who do become sick as a result of workplace exposure, Workers’ Compensation benefits should be mandated, using a conclusive presumption.**

- Provide for strong whistleblower protections to protect and encourage workers’ ability to report hazardous conditions and noncompliance. It should ensure vigorous protection and defense of whistleblowers who report dangerous workplace conditions that threaten to infect, make ill, or cause death from exposure to SARS-CoV-2.
- Provide strong worker rights to refuse dangerous work when adequate safety protections are not provided, with no loss of pay.
- Require employers to inform all co-workers who have been in close contact with a worker known to have COVID-19 infection of their possible exposure to COVID-19 in the workplace, keeping the infected worker’s identity confidential in accordance with the American with Disabilities Act. All workers who have been exposed to SARS-CoV-2 should have a right to quarantine with pay for 14 days. We also need clear “return-to-work” standards for when employees can return to work after exposure or illness.
- Prohibit employers from enacting or continuing incentives or bonuses for not using sick time, for reporting to work for a certain number of days or weeks in a row, or related policies that discourage workers from being absent from work and from utilizing sick time.
- Mandate that employers discontinue production and service quotas that promote speed-up and discourage safe work practices as they prevent work from being performed in a manner that will minimize possible SARS-CoV-2 transmission. Any rule or practice that limits workers’ time for proper handwashing and sanitation should be eliminated.
- Workers in high-risk categories whose work remains reduced, suspended, or eliminated because of the COVID crisis must receive paid leave during the time they are not working. This must include all public and private sector workers including independent contractors, persons performing work for an employer through a temporary services or staffing agency, and undocumented workers.
- Workers who have quit their jobs to protect themselves or were fired for refusing to work under what they reasonably believed were dangerous conditions should be granted “just cause” and deemed eligible for unemployment insurance. Furthermore, such “good cause quits” under UI should include a worker’s need to quit to care for quarantined or sick family or household members.
- Workers’ Compensation benefits should be mandated, using a conclusive presumption, for *all* workers who are exposed to other workers or the public at the workplace and become infected with COVID-19.
- Provide for workers’ rights to job retention and protected right to return to work must be provided. For workers who have been laid off due to pandemic-related business location closure, ensure that they have the right to return to their job once the business or location resumes operations. In the case of a layoff due to lack of work resulting from the pandemic, such workers should be given priority to return to their position once re-hiring commences. Worker retention policies must include the protection of workers’ jobs in the event of subcontracting, bankruptcy reorganization, or a change in ownership that occurs as a result of the pandemic.
  - Expand anti-discrimination, disability, and accommodation protection for workers who have recovered but have sustained health impairments, for pregnant workers, and those who are in high risk categories (older workers, workers with underlying conditions, workers with impaired immune systems).

**Workplace exposure is a major way our residents are becoming ill and spreading the virus to their families and communities. Before we move to Phase 2, we must begin collecting and analyzing data on the occupation and industry of COVID-19 cases and develop procedures to investigate workplace outbreaks and close businesses due to COVID-19 infections and outbreaks when needed.**

- By looking at patterns of COVID-19 across industry and occupation, it is possible to assess potential risks faced by different worker groups. The statewide public health surveillance system should collect information about whether individuals with COVID worked outside of their home in the 14 days prior to disease onset and their occupation, industry, as well as employer name and job site location. These data will allow us to assess which jobs in the economy may put workers at greater risk of illness and use that information to improve workplace protection. They will also allow us to identify employers who are failing to implement adequate steps, such as paid sick leave for isolation and quarantine, as well as adequate ventilation, social distancing, and paid time for hand washing.
- The statewide public health surveillance system should also collect information on race and ethnicity of all COVID-19 cases to describe the unequal burden of COVID-19 on communities of color and reveal how work is contributing to that disproportion.
- Contact tracers should collect data on whether the individual was employed outside their home in the 14 days prior to disease onset and occupation, industry, as well as employer name and site location. Protocols should be implemented to allow for early identification of workplace clusters or outbreaks. Contact tracing protocols should be multilingual and designed to protect the confidentiality of infected workers.
- Investigate outbreaks or clusters of COVID-19 in workplaces to assure that interventions to prevent or reduce exposures are implemented. Seek input from affected workers and union representatives. Establish procedures for closing workplaces due to COVID-19 infections and outbreaks.
- Ensure that workers have access to free, accessible, reliable, and rapid COVID-19 testing to take place on paid time. Results of the test should be communicated in writing, in multiple languages, and include information on how to seek medical care. If a worker is found to have COVID-19, local public health authorities and/or the employer should ensure that all worker contacts are tested and quarantined.
- Prevent stigma and discrimination by ensuring that determinations of risk are not based on race or country of origin, and that the confidentiality of those with confirmed COVID-19 is maintained.

